# Primary aldosteronism: pharmacotherapy *vs* surgery *vs* embolization efficacy – systematic review and network meta-analysis of studies predominantly conducted in China

**DOI:** 10.3389/fendo.2025.1645279

**Published:** 2025-11-17

**Authors:** Gang Li, Wenping Xu, Changjun Li, Peng Hu, Siqin Qin

**Affiliations:** 1 Department of Urology, Mianzhu City People’s Hospital, Mianzhu, Sichuan, China; 2 Department of Urology, Cangxi County People’s Hospital, Guangyuan, Sichuan, China; 3 Department of Urology, Zitong County People’s Hospital, Mianyang, Sichuan, China; 4 School of Clinical Medical, Chengdu Medical College, Chengdu, Sichuan, China

**Keywords:** primary aldosteronism, adrenalectomy, radiofrequency ablation, super selective adrenal artery embolization, meta-analysis

## Abstract

**Background:**

Primary aldosteronism (PA) is one of major cause of resistant hypertension. This study compares the efficacy and safety of current treatments, including pharmacotherapy, adrenalectomy, and minimally invasive techniques, to guide clinical practice.

**Method:**

We systematically searched Embase, PubMed, Cochrane, Web of Science, CNKI, Wanfang, and VIP from inception to August 13, 2024, for randomized controlled trials and cohort studies involving adult patients with PA and hypertension. Reporting quality of the included studies was assessed using the Cochrane Risk of Bias 2 tool for RCTs and the Newcastle-Ottawa Scale (NOS) for cohort studies. Data analysis was performed using R 4.3.3 and STATA 15.0.

**Results:**

This study included 17 articles involving 1,496 patients, of which 13 studies (76%) were conducted in China. Meta-analysis showed that for systolic blood pressure (SBP), unilateral total adrenalectomy and renal nerve denervation (TADR+RND) was most effective (WMD = -12.53, 95% Crl -15.18 to -9.90). For diastolic blood pressure (DBP), Partial adrenalectomy (PADR) (WMD = -9.31, 95% Crl -12.97 to -5.68). PADR also maintained serum potassium levels effectively (0.64, 95% Crl 0.52 to 0.75). Among pharmacological treatments, mineralocorticoid receptor antagonists and irbesartan (MRAs+IRB) had the greatest antihypertensive effect (SBP: WMD = -18.90, 95% Crl -29.20 to -8.55; DBP: WMD = -22.14, 95% Crl -31.81 to -12.50). Mineralocorticoid receptor antagonists did not significantly reduce plasma aldosterone concentration (PAC), consistent with their known feedback-related tendency to increase PAC.

**Conclusions:**

This study showed TADR + RND and MRAs + IRB had best efficacy in surgical and pharmacological treatments, respectively, but 76% of the included studies were conducted in China, which may affect the generalizability of the findings. Therefore, the results need further validation.

## Graphical Abstract

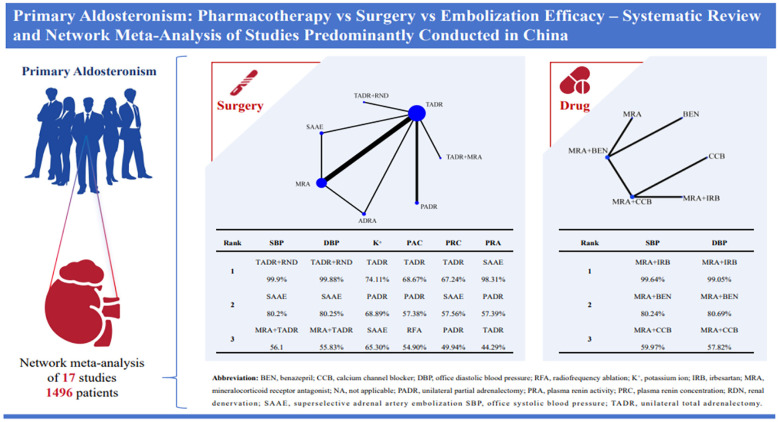

## 1 Introduction

Primary aldosteronism (PA) is considered one of the leading causes of resistant hypertension (HT), accounting for approximately 20% of resistant HT cases according to recent epidemiological studies (1), and is also an important contributor to cardiovascular events. Although some PA cases are detected due to hypokalemia in patients, the true prevalence of PA is often underestimated because of the high degree of similarity in clinical symptoms between PA and essential HT (2). It was found that patients with PA had higher cardiovascular morbidity and mortality than patients with essential HT of the same age and gender and patients with the same increase in blood pressure (3). A further study found that cardiovascular risk in patients with PA was related to not only HT itself but also other metabolic abnormalities caused by PA, such as insulin resistance, inflammation and oxidative stress (4). These factors may work together to accelerate the development of atherosclerosis, and increase the risk of myocardial infarction, heart failure and stroke. Therefore, timely diagnosis and effective treatment of PA are critical.

Current treatments for PA include pharmacotherapy, adrenalectomy, radiofrequency ablation (RFA), and super selective adrenal artery embolization (SAAE) (1), According to current and previous Endocrine Society guidelines (5, 6). treatment recommendations depend on the lateralization status of aldosterone excess: unilateral total adrenalectomy is the preferred option for unilateral PA, whereas medical therapy with mineralocorticoid receptor antagonists (MRAs) is the mainstay for bilateral disease, undetermined subtype, or when surgery is not feasible or not desired. Pharmacotherapy usually includes the use of MRAs such as spironolactone and eplerenone, which are effective in controlling blood pressure and correcting electrolyte imbalance. In addition, MRAs increase plasma renin concentration and have been shown to reduce the risk of cardiovascular and renal complications in patients with PA (7). However, because patients with PA may take these medications for a long period of time, they can face certain risks of side effects, such as gynecomastia, erectile dysfunction, and mastalgia (2). Therefore, to reduce the side effects of single drugs and enhance the antihypertensive effect, they usually receive MRAs plus other antihypertensive drugs. However, the effects of combining MRAs with different classes of antihypertensive drugs (e.g., ACE inhibitors, ARBs, or calcium channel blockers) remain unclear, particularly with respect to blood pressure control, biochemical normalization, and adverse events, and there are notable inter-individual variations in treatment response.

Adrenalectomy, as a curative option for unilateral PA, removes aldosterone-producing tissue and improves both blood pressure and metabolic outcomes (9). Minimally invasive approaches, including laparoscopic and robotic-assisted surgery, have further reduced complications and recovery time compared with traditional open surgery (10). Other emerging strategies such as renal denervation (RDN), radiofrequency ablation (RFA), and super selective adrenal artery embolization (SAAE) are considered non-standard or investigational in current guidelines. For example, SAAE, as minimally invasive treatments, offer alternative treatment options for patients with PA, particularly in cases of lateralized disease when conventional adrenalectomy is not feasible or not desired. RDN may inhibit excessive activation of the renin-angiotensin-aldosterone system (RAAS) by reducing renal sympathetic activity, thereby lowering blood pressure (11). RFA and SAAE, as minimally invasive treatments, can reduce aldosterone secretion locally while preserving part of adrenal function (12, 13). Although these therapies may provide short-term improvements in blood pressure and biochemical markers, long-term efficacy and safety remain to be established.

Although existing studies have explored the efficacy of these treatments, there is relatively limited evidence comparing them directly, and there is a lack of uniform evaluation criteria. Therefore, this systematic review and network meta-analysis (NMA) was conducted to synthesize currently available evidence and comprehensively assess and compare the efficacy of the above treatments in controlling blood pressure and improving biochemical indicators in patients with PA, with a view to providing a more solid scientific rationale for clinical decision-making.

## 2 Methods

This systematic review and meta-analysis followed the Preferred Reporting Items for Systematic Reviews and Meta-Analyses (PRISMA) 2020 guidelines (14) (**Supplementary Table S1**). The study protocol was already registered in the International Prospective Register of Systematic Reviews (PROSPERO: CRD42024600125).

### 2.1 Data sources and search strategy

We systematically searched Embase, PubMed, Cochrane, Web of Science, China National Knowledge Infrastructure (CNKI), Wanfang, and VIP from inception to August 13, 2024, using primary aldosteronism as a keyword. A search strategy for various databases is shown in **Supplementary Table S2**. In addition, to ensure a comprehensive search, a manual search was conducted by checking the reference lists in previous systematic reviews to identify relevant studies that may have been missed. All retrieved references were downloaded and imported into EndNote 21 for standardized management to facilitate subsequent screening, review and citation.

### 2.2 Inclusion and exclusion criteria

Inclusion criteria were as follows: (1) study designs included randomized controlled trials and cohort studies; (2) study subjects were male adults or non-pregnant patients (age ≥18 years) diagnosed with PA and suffering from hypertension; (3) study subjects had no history of accelerated or malignant hypertension, sex hormone therapy or renal hypofunction; (4) studies reported endpoints include SBP, DBP, blood potassium level, serum aldosterone concentration (SAC), plasma renin concentration (PRC), and plasma renin activity (PRA); (5) studies written in English or Chinese. Those studies that were not eligible for inclusion were excluded. Where multiple reports were published from the same study cohort, we included only the studies with the most detailed information and sample sizes.

### 2.3 Risk of bias assessment

Two authors (Li and Xu) assessed the quality of each included randomized controlled trial (RCT) using a Cochrane risk of bias assessment tool (RoB2) (15). For cohort studies, the two authors independently assessed them using the Newcastle-Ottawa Quality Assessment Scale (NOS) (http://www.ohri.ca/programs/clinical_epidemiology/oxford.asp.). Discrepancies were resolved by discussion to reach a consensus. The results of the quality assessment were used to evaluate the reliability of the included studies, and sensitivity analysis was performed.

### 2.4 Data extraction

In this systematic review, the data extraction process followed strict scientific guidelines to ensure the accuracy and consistency of data. All data were extracted separately by Li and Xu to minimize bias that could be introduced by a single person extracting data. Discrepancies were resolved by discussion. Information obtained from each study included the last name of the first author, year of publication, country, intervention, study type, follow-up time, number of participants, gender, age, and information on relevant outcome measures.

The extracted outcome data were the mean changes from baseline to the last available follow-up, together with the corresponding standard deviation (SD). We prioritized intention-to-treat data when primary studies reported per-protocol and intention-to-treat analyses. When primary studies reported the mean difference (MD) and standard error or did not report the MD, we used appropriate mathematical transformations to obtain the required data, as detailed in **Supplementary Material**. When data at multiple time points were reported in primary studies, this systematic review used data from uniform or scientifically close time points for analysis to ensure consistency of comparisons and reduce potential bias caused by different follow-up times. When the outcome data of interest were missing, we contacted the corresponding study authors to obtain the data.

Primary outcome measures were office systolic and diastolic blood pressure, and plasma potassium ion concentration. Secondary outcome measures were serum aldosterone concentration, plasma renin concentration, and plasma renin activity.

### 2.5 Data analysis

The main effect size was reported as MD or standardized mean difference (SMD) to measure the efficacy of different interventions in lowering blood pressure and improving biochemical indicators. A 95% confidence interval (CI) was calculated along with the effect size. Calculations were performed utilizing the “rjags” package in the R software. A network diagram was created through a Bayesian simulation model to visualize the relationships among various interventions. In the diagram, a node represents an intervention, a line represents a direct comparison, as well as the thickness of the line and the size of the node are proportional to the number of studies and the number of participants involved in direct comparison. Considering that the NMA was based on homogeneity, similarity and consistency assumptions (16), the consistency test and homogeneity test were further performed.

Inconsistency was identified by two methods. First, the SDs of random effects were calculated under the consistency and inconsistency models to determine whether inconsistency existed among interventions. If the SDs of random effects were the same under both the models, it indicated good consistency among interventions. Second, the *P*-values calculated in node-splitting analysis were checked to determine the applicability of the models. Node-splitting analysis, another method to assess inconsistency in a network meta-analysis, was used to assess the consistency of direct and indirect evidence on split nodes. If the *P*-value for all direct and indirect evidence comparisons was more than 0.05, a network meta-analysis was performed using the consistency model (17) If there was no closed loop in the network diagram, the consistency model would be used to summarize the relative efficacy of the included interventions. Heterogeneity among studies was assessed using I^2^ and the Cochran’s Q test. I^2^ ≥25% indicated low heterogeneity. I^2^ ≥50% indicated moderate heterogeneity. I^2^ ≥75% indicated high heterogeneity. If I^2^ >50%, a random-effects model would be used; otherwise, a fixed-effects model would be used (18). The rank order of different treatment options was assessed using the surface under the cumulative ranking curve (SUCRA). A higher SUCRA value denoted better efficacy. In addition, publication bias test and sensitivity analysis were performed to ensure the accuracy and reliability of the analysis results.

## 3 Results

### 3.1 General characteristics

**Figure 1:**
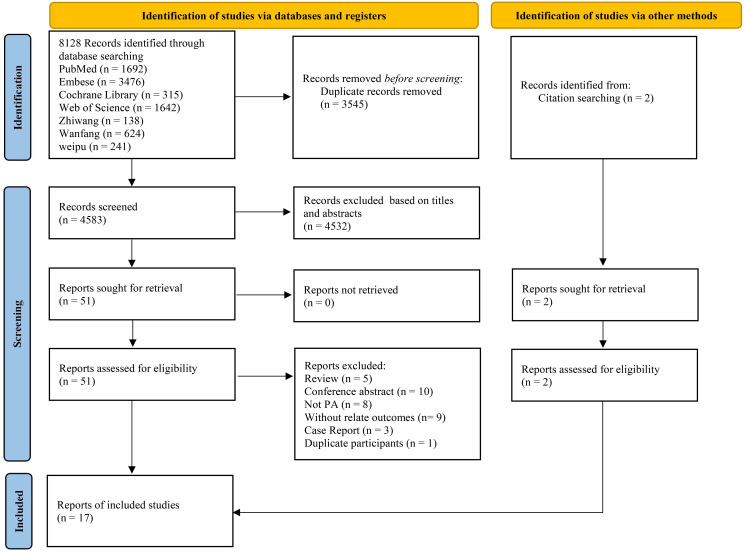
Literature screening flow chart.

A total of 8128 studies were retrieved, with 3545 of them remaining after automatic removal of duplicates by EndNote 21. After further screening of titles and abstracts, a total of 51 studies were advanced to full-text review. Thirty-six of these did not satisfy the inclusion criteria, and two additional studies were located through hand-searching. Finally, 17 with 1496 participants were ultimately included in the analysis (19–35), as detailed in **Figure 1**. Of these studies, 13 (76%) were conducted in China, 14 (82%) were RCTs, and the remaining 3 were cohort studies. This study involved a total of six surgical treatments and four pharmacologic treatments. Surgical treatments included RFA, unilateral partial adrenalectomy (PADR), unilateral total adrenalectomy (TADR), SAAE, TADR plus MRAs, and TADR plus RDN. Pharmacologic treatments involved MRAs, calcium channel blockers (CCBs), benazepril (BEN), irbesartan (IRB) and their combinations. The main characteristics of the included studies are shown in **Table 1**. Detailed information on drug regimens and dosages, when available, is summarized in **Supplementary Table S3**.

**Table 1:** Characteristics of included studies.

Author	Year	Country	Design	Treatment	Sample size	Age (Years) (mean ± SD)	Sex (male/female)	Outcomes	Duration (Months)
Castro et al (19)	2022	Spain	Cohort	MRA	168	54.7 ± 12.5	98/70	SBP, DBP, K^+^, SAC, PRC, PRA	23.6
				TADR	100	52.7 ± 9.4	46/54		
Chen et al (20)	2014	China	RCT	PADR	16	48.5 ± 10.9	7/9	SBP, DBP, K^+^, SAC, PRC	12
				TADR	47	48.7 ± 11.3	21/26		
Chen et al (21)	2021	China	RCT	TADR	39	49.4 ± 10.2	26/13	SBP, DBP, K^+^, SAC, PRA	6
				MRA	28	48.8 ± 11.5	22/6		
Dang et al (22)	2022	China	RCT	MRA	30	56.84 ± 6.22	15/15	SBP, DBP	3
				BEN+MRA	30	56.84 ± 6.22	14/16		
Fu et al (23)	2011	China	RCT	PADR	104	43 ± 5.8	45/59	SBP, DBP, K^+^, SAC, PRA	96
				TADR	108	41 ± 7.8	48/60		
Liu (24)	2018	China	RCT	MRA+CCB	20	NA	NA	SBP, DBP	3
				BEN+MRA	20	NA	NA		
Liu et al (25)	2021	China	RCT	TADR	30	50.3 ± 9.7	13/17	SBP, DBP	6
				TADR+RDN	30	50.0 ± 10.9	11/19		
Indra et al (26)	2015	Czech Republic	RCT	MRA	16	51.0 ± 7.0	11/5	SBP, DBP, K^+^	64
				TADR	15	49.0 ± 11.0	9/6		
Puar et al (27)	2020	Singapore	Cohort	TADR	86	51.0 ± 10.1	49/37	SBP, DBP, K^+^,	120
				MRA	68	55.0 ± 9.1	46/22	SAC, PRA	
Qi et al (28)	2021	China	RCT	TADR	41	33.6 ± 7. 2	20/21	SBP, DBP, K^+^, SAC, PRC	6
				SAAE	41	32.2 ± 5.6	22/19		
Tan et al (29)	2021	China	RCT	CCB	33	45.84 ± 2.31	13/20	SBP, DBP, SAC	6
				MRA+ CCB	33	45.92 ± 1.82	10/23		
Wu (30)	2017	China	RCT	BEN	40	61.1 ± 2.6	28/12	SBP, DBP	6
				BEN+MRA	40	61.1 ± 2.1	30/10		
Yang et al (31)	2016	China	RCT	RFA^1^	7	NA	4/3	SBP, DBP, K^+^, SAC	6
				TADR	18	NA	7/11		
Zhao et al (32)	2021	China	RCT	MRA	25	NA	NA	SBP, DBP, K^+^, SAC, PRC	
				RFA^2^	26	NA	NA		
Zhang et al (33)	2021	Australia	Cohort	TADR+MRA	73	NA	48/25	SBP, DBP, K^+^	24
				TADR	23	NA	8/15		
Zhou et al (34)	2023	China	RCT	MRA	30	56.0 ± 15.8	6/24	SBP, DBP, K^+^, SAC, PRC	36
				SAAE	29	55.5 ± 12.1	10/19		
Zhu et al (35)	2017	China	RCT	MRA+ CCB	40	NA	NA	SBP, DBP	3
				MRA+ IRB	42	NA	NA		

^1,^ CT-guided catheter puncture approach; ^2,^ CT-guided percutaneous approach; BEN, benazepril; CCB, calcium channel blocker; DBP, office diastolic blood pressure; RFA, radiofrequency ablation; K+, potassium ion; IRB, irbesartan; MRA, mineralocorticoid receptor antagonist; NA, not applicable; PADR, unilateral partial adrenalectomy; PRA, plasma renin activity; PRC, plasma renin concentration; RDN, renal denervation; SAAE, super selective adrenal artery embolization SBP, office systolic blood pressure; TADR, unilateral total adrenalectomy.

### 3.2 Quality assessment

RoB2 was used to assess the quality of the fourteen RCTs. Of them, five studies were assessed as having a moderate risk of bias, and nine studies were assessed as having a high risk of bias. The results of the assessment may be partly attributed to the surgical treatments and pharmacological treatments in the study design. These interventions required participants to sign informed consent forms prior to participation to fulfill ethical review requirements, which, meanwhile, limited the possibility of implementation of blinding in the studies, thus affecting the quality scores for the interventions in RoB2 assessment, The results are shown in **Supplementary Figure S1**. In addition, the NOS was used to assess the quality of the three cohort studies. All of them were assessed as high-quality studies. The results are shown in **Supplementary Table S4**.

### 3.3 Primary outcomes

#### 3.3.1 Systolic blood pressure

**Figure 2:**
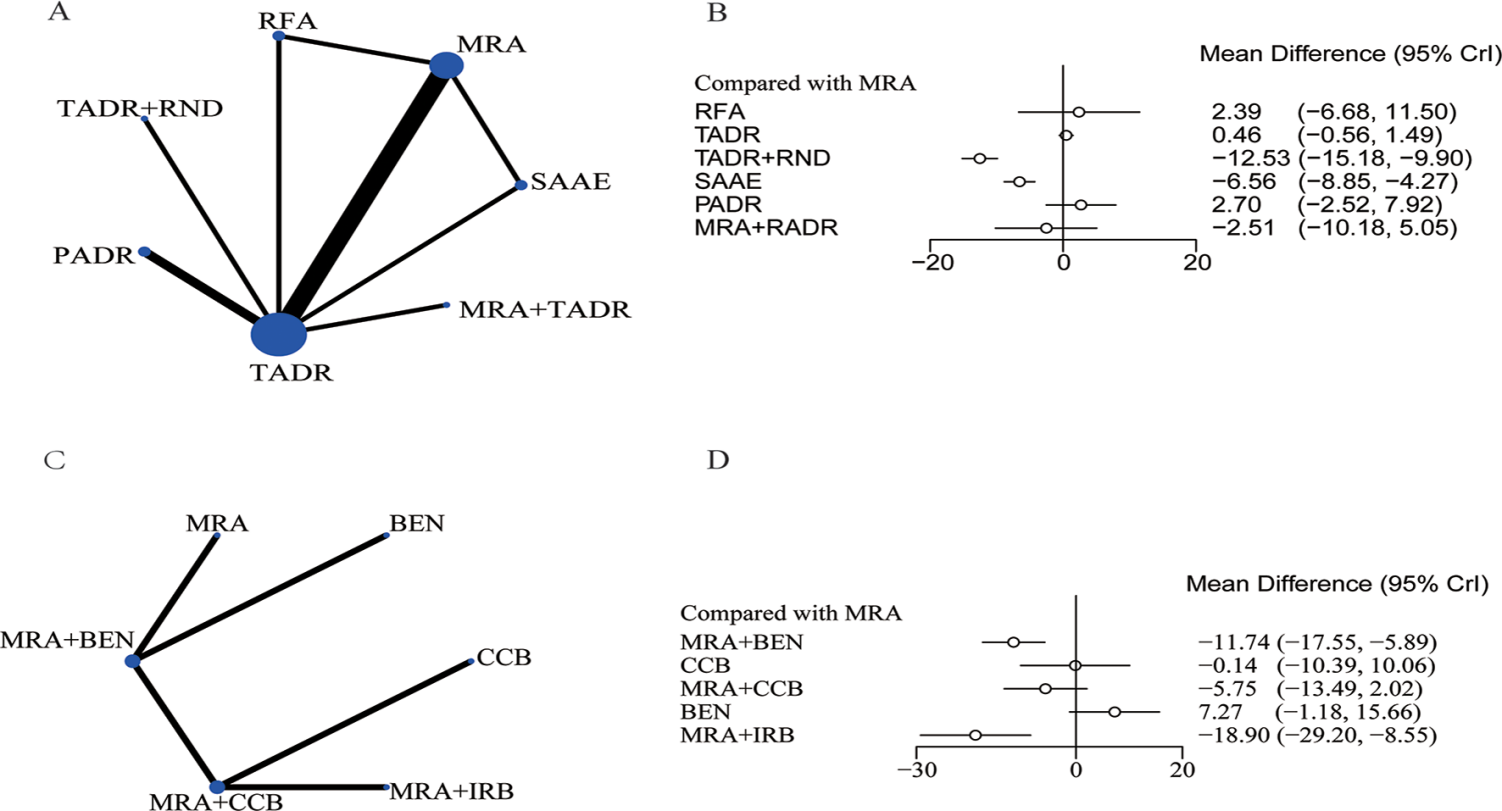
Systolic blood pressure, **(A)** Network Diagram; **(B)** Forest Plot of Relative Efficacy; **(C)** Network Diagram (drugs); **(D)** Forest Plot of Relative Efficacy (drugs).

The network diagram for surgical and pharmacological treatments is shown in **Figure 2A**. They included seven treatment options: TADR, TADR + MRAs, PADR, MRAs, RFA, SAAE, and TADR + RND. Compared with MRAs, TADR + RND showed the most significant efficacy in reducing SBP (WMD = -12.53;.95% Crl: -15.18, -9.90), followed by SAAE (WMD = -6.56; 95% Crl: -8.85, -4.27), as detailed in **Figure 2B**. In addition, different pharmacologic treatments were compared. A network diagram for them is shown in **Figure 2C**. Compared with MRAs, MRAs + IRB showed the most significant efficacy in reducing SBP (WMD = -18.90; 95% Crl: -29.20, -8.55), followed by MRAs + benazepril(BEN) (WMD = -11.74; 95% Crl: -17.55, -5.89), as detailed in **Figure 2D**. The NMA estimates for the relative efficacy of all interventions in terms of SBP reduction are detailed in **Supplementary Tables S5**, **6**.

#### 3.3.2 Diastolic blood pressure and potassium ion

**Figure 3:**
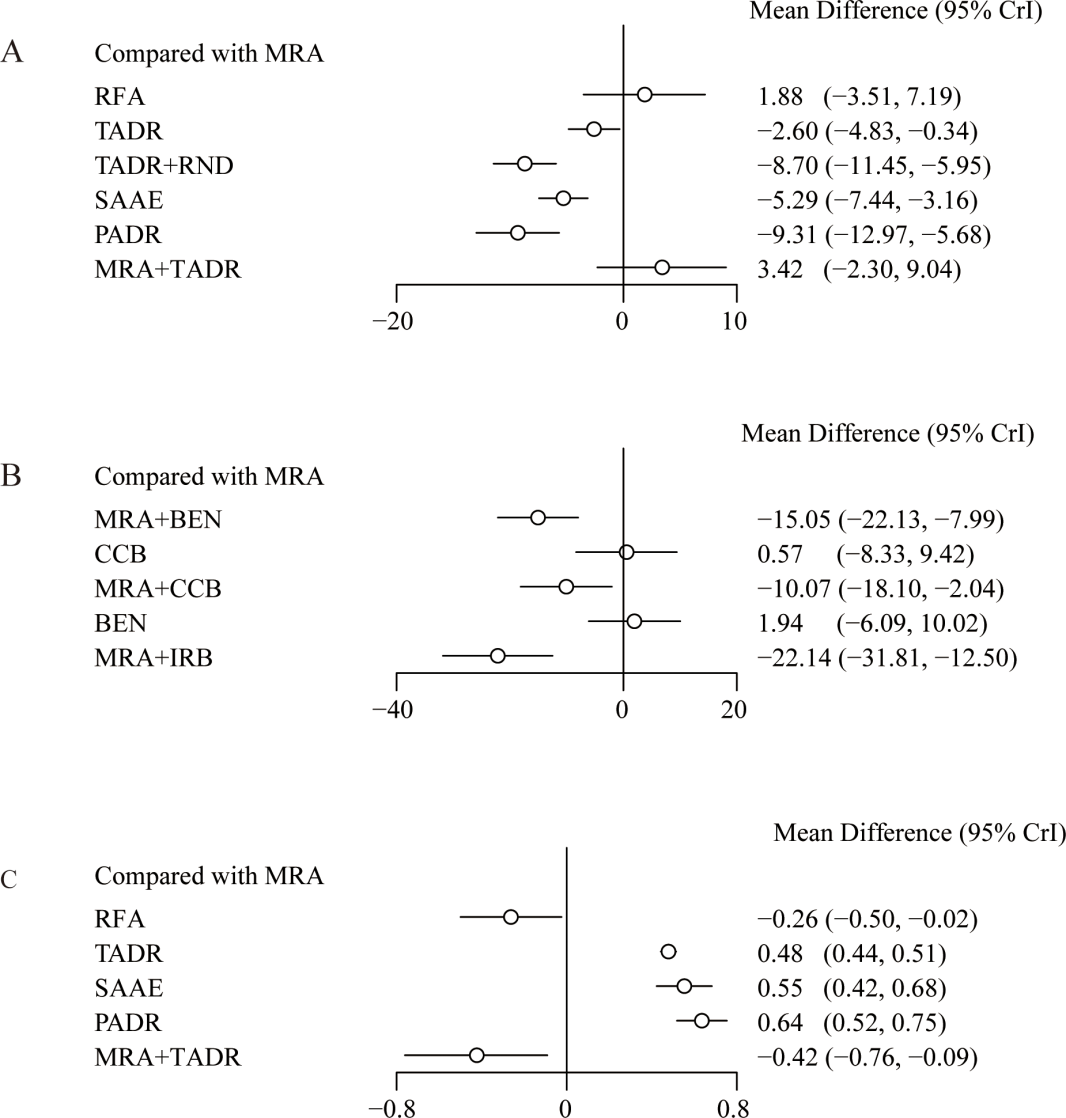
Effect sizes of various treatments relative to MRAs, **(A)** Diastolic Blood Pressure; **(B)** Diastolic Blood Pressure (drugs); **(C)** Plasma K^+^ Levels.

Compared with MRAs, PADR had the most significant efficacy in reducing DBP (WMD = -9.31; 95% Crl: -12.97, -5.68), followed by TADR + RND (WMD = -8.70; 95% Crl: -11.45, -5.95), as detailed in **Figure 3A**. In addition, the comparison of pharmacologic treatments showed that compared with MRAs, MRAs plus IRB had the most significant efficacy in reducing DBP (WMD = -22.14, 95% Crl: -31.81, -12.50), followed by MRAs plus BEN (WMD = -15.05, 95% Crl: -22.13, -7.99), as detailed in **Figure 3B**. The NMA estimates for the relative efficacy of all interventions in terms of DBP reduction are detailed in **Supplementary Tables S5**, **6**. In maintaining the K^+^ level, PADR, SAAE and TADR all showed significant efficacy, with PADR having the most significant efficacy (WMD=0.64; 95% Crl: 0.52, 0.75), as detailed in **Figure 3C**. The NMA estimates for the relative efficacy of all interventions in terms of K^+^ elevation are detailed in **Supplementary Table S7**.

#### 3.3.3 Secondary outcomes

**Figure 4:**
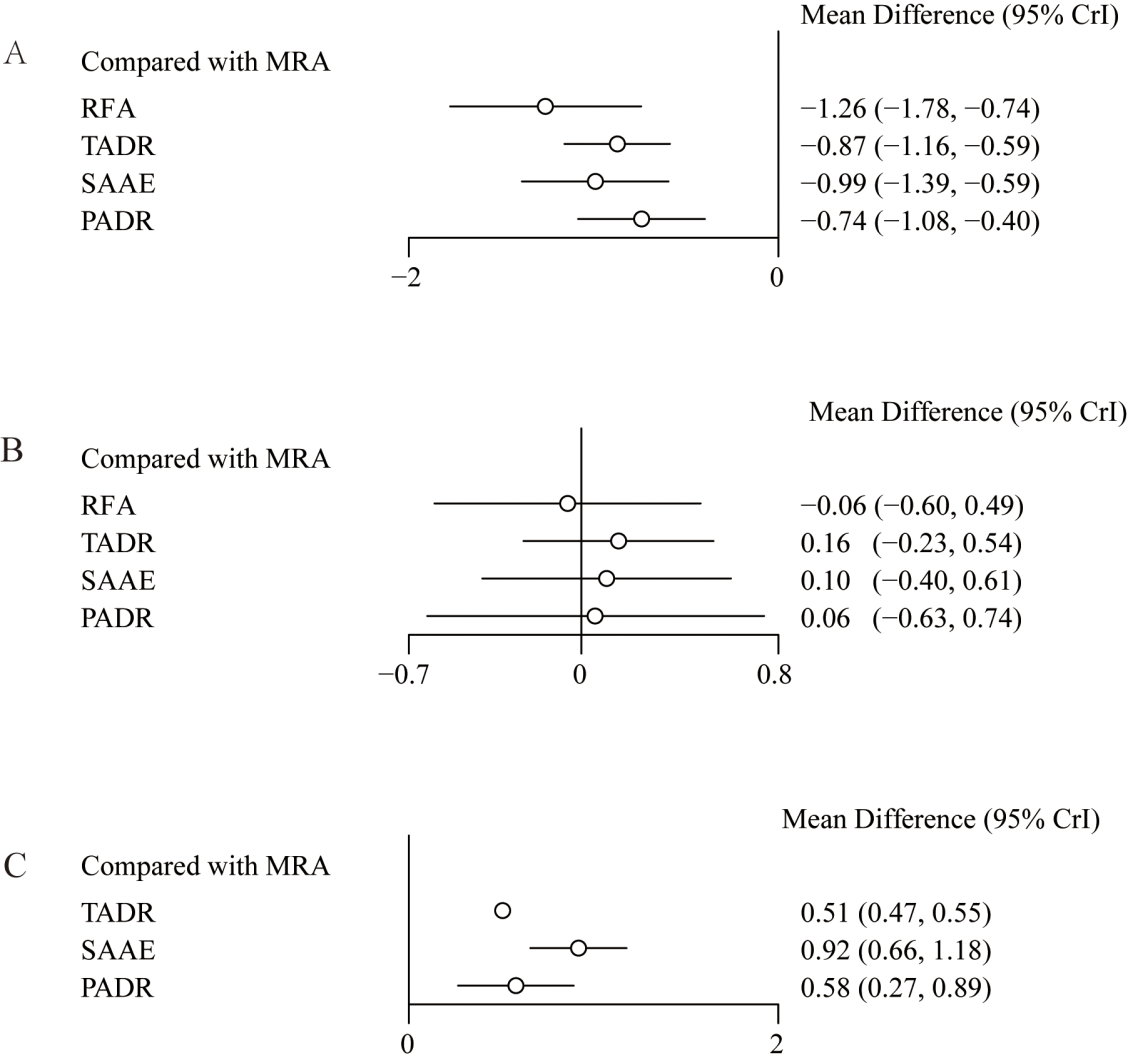
Effect sizes of various treatments relative to MRAs, **(A)** Plasma Aldosterone Concentration (PAC); **(B)** Plasma Renin Concentration (PRC); **(C)** Plasma Renin Activity (PRA).

Considering the pathological characteristics of PA, we further performed a meta-analysis for plasma aldosterone concentration (PAC), plasma renin concentration (PRC) and plasma renin activity (PRA). Five studies reported PAC, the forest plot (**Figure 4A**) shows comparisons against MRAs, but such comparisons should be interpreted with caution because MRAs are known to increase aldosterone levels. To provide clinically meaningful insight, we further examined the league table estimates (**Supplementary Table S7**), which allowed co\mparisons between surgical strategies. Although RFA tended to show a greater reduction in PAC compared with TADR, SAAE, and PADR, these differences were not statistically significant.

Five studies reported changes in PRC, the results and showed no statistically significant differences between MRAs and the other interventions, as detailed in **Figure 4B**. In addition, four studies reported changes in PRA and showed that compared with MRAs, TADR, SAAE and PADR all significantly increased PRA, with SAAE having the most significant efficacy (WMD=0.92, 95% Crl: 0.66, 1.18), as detailed in **Figure 4C**. The NMA estimates for the relative efficacy of all interventions in terms of PRC and PRA elevation are detailed in **Supplementary Table S8**.

#### 3.3.4 SUCRA value

The ranking of different interventions according to SUCRA values for efficacy is shown in **Table 2**. Overall, of surgical interventions, TADR plus RND had the best efficacy in lowering blood pressure, followed by SAAE. In addition, TADR and SAAE showed good efficacy in maintaining serum K+ levels, increasing PRC, and decreasing PAC. Of the pharmacological treatments, MRAs plus IRB had the most significant antihypertensive efficacy, and MRAs plus other antihypertensive drugs had significantly better efficacy than MRAs alone or a single antihypertensive drug.

**Table 2:** SUCRA Values and treatment efficacy rankings.

Rank	Outcomes							
	SBP	DBP	SBP (drug)	DBP (drug)	K^+^	PAC	PRC	PRA
1	TADR+RND 99.9%	TADR+RND 99.88%	MRA+IRB 99.64%	MRA+IRB 99.05%	TADR 74.11%	TADR 68.67%	TADR 67.24%	SAAE 98.31%
2	SAAE 80.2%	SAAE 80.25%	MRA+BEN 80.24%	MRA+BEN 80.69%	PADR 68.89%	PADR 57.38%	SAAE 57.56%	PADR 57.39%
3	MRA+TADR 56.1%	MRA+TADR 55.83%	MRA+CCB 59.97%	MRA+CCB 57.82%	SAAE 65.30%	RFA 54.90%	PADR 49.94%	TADR 44.29%
4	MRA 43.33%	MRA 43.47%	MRA 25.01%	MRA 30.29%	MRA 45.29%	SAAE 49.23%	MRA 39.48%	MRA 0.01%
5	TADR 31.28%	TADR 31.10%	CCB 22.11%	CCB 29.74%	MRA+TADR 37.02%	MRA 19.82%	RFA 35.79%	NA
6	RFA 23.42%	RFA 23.66%	BEN 13.03%	BEN 2.42%	RFA 9.40%	NA	NA	NA
7	PADR 15.83%	PADR 15.83%	NA	NA	NA	NA	NA	NA

BEN, benazepril; CCB, calcium channel blocker; DBP, office diastolic blood pressure; RFA, radiofrequency ablation; K+, potassium ion; IRB, irbesartan; MRA, mineralocorticoid receptor antagonist; NA, not applicable; PADR, unilateral partial adrenalectomy; PRA, plasma renin activity; PRC, plasma renin concentration; RDN, renal denervation; SAAE, superselective adrenal artery embolization SBP, office systolic blood pressure; TADR, unilateral total adrenalectomy.

## 4 Discussion

This study compared the efficacy of pharmacotherapy, TADR, PADR RFA, and SAAE in lowering blood pressure and improving biochemical indicators in patients with PA. The results of this study showed that TADR plus RND had the best antihypertensive efficacy in surgical treatment, whereas MRAs plus IRB showed the best efficacy among pharmacological treatments.

An in-depth discussion of the indications, mechanisms of action, advantages and limitations of different treatments is crucial for the development of individualized treatment strategies. Surgical treatment is mainly suitable for patients with unilateral adrenal adenomas or unilateral adrenal hyperplasia, especially when pharmacotherapy is difficult to effectively control blood pressure or biochemical abnormalities. Unilateral adrenalectomy significantly lowers blood pressure and corrects hypokalemia by removing adrenal tissue that overproduces aldosterone, thereby addressing the root cause of the problem (36).In addition, Ma, et al. suggested that surgery may improve diastolic blood pressure control in patients with unilateral lesions, especially those who do not respond to pharmacotherapy (38).

Different surgical procedures have their own advantages and disadvantages depending on the patient’s clinical manifestations and lesions. TADR plus RND is indicated for patients with blood pressure difficult to control and concomitant serious hypokalemia. In this study, TADR plus RND had the best antihypertensive effect, and its dual mechanism of action may explain its significant antihypertensive efficacy. TADR reduces aldosterone secretion by excision of lesions, whereas RND reduces the influence of sympathetic activity on blood pressure by the autonomic nervous system (39, 40). This finding echoes a study by Camafort, et al., which shows that an intervention for sympathetic denervation is effective in reducing blood pressure in patients with refractory hypertension (41). In addition, a previous study has also pointed out that overactivity of the sympathetic nervous system (SNA) in patients with PA is an important factor in the maintenance of hypertension, and multiple mechanisms of action may be the key to the efficacy of unilateral adrenalectomy plus RND (42).

SAAE is a minimally invasive option to reduce local aldosterone secretion while preserving partial adrenal function. It is suitable for patients who require postoperative biochemical monitoring or who hope to preserve partial adrenal function in part of the adrenal gland (43). This study showed that SAAE was effective in not only controlling blood pressure but also maintaining the stability of renin levels in the body. Consistent with this study, a study by Wang, et al. showed that SAAE was able to achieve significant antihypertensive efficacy while preserving adrenal function, and the incidence of postoperative complications was low (12). However, the long-term efficacy of SAAE compared with other surgical procedures should be further investigated, especially in direct comparison with aggressive surgical approaches such as unilateral adrenalectomy or RND.

RFA, due to its minimally invasive nature, short time to recovery and low surgical risk, offers a relatively safe option for patients of advanced age or with a combination of chronic diseases who cannot tolerate traditional open or laparoscopic surgery (8). Liu et al. further conducted a randomized trial comparing renal denervation (RDN) from the adventitia of the renal artery plus TADR with TADR alone in patients with resistant hypertension caused by unilateral APA. At 36 months of follow-up, the RDN group showed a significantly greater reduction in office systolic blood pressure (42.2 ± 21.6 mmHg) compared with the TADR-only group (29.8 ± 13.5 mmHg, p = 0.029), with no serious procedure-related complications reported (39). These findings suggest that RDN combined with TADR may enhance the antihypertensive efficacy of surgery in selected patients, without compromising long-term safety (25).

Previous studies have shown that patients with bilateral hyperplasia have good outcomes after treatment with drugs, especially MRAs, which are effective in controlling blood pressure and improving biochemical indicators (1). Despite their widespread use in patients with PA, MRAs may pose risks, particularly hyperkalemia and renal impairment. As MRAs block aldosterone’s effect on potassium excretion, some patients, especially those with renal impairment or those using other potassium-sparing medications, may experience hyperkalemia, which in severe cases can lead to fatal arrhythmias (7).

To reduce the side effects of MRAs, they are often combined with other antihypertensive drugs to achieve better therapeutic outcomes and reduce adverse reactions to monotherapy. For example, MRA combined with angiotensin II receptor blockers such as IRB is more effective in controlling blood pressure by doubly blocking different components of the RAAS system, while reducing the risk of hyperkalemia posed by MRAs (39). This combination therapy regimen can not only enhance antihypertensive efficacy but also reduce side effects of MRAs, such as gynecomastia, menstrual disorders and other hormone-related problems, by reducing the dose of MRAs (39). In addition, IRB plus MRAs can further improve biochemical abnormalities, for example, lower aldosterone levels and relieve hyporeninemia, thus enhancing overall patient outcomes (44). However, drug combination therapy may also bring side effects such as hyperkalemia and impaired renal function, which need to be closely monitored especially in patients with renal insufficiency.

The choice of surgery and pharmacotherapy depends on the pathological type and clinical manifestations in patients. For patients with unilateral adenoma or hyperplasia, surgery is the curative option, whereas pharmacotherapy is more suitable for patients with bilateral lesions or those who cannot tolerate surgery (45). It is worth noting that patients who did not undergo adrenal venous sampling (AVS) may be confused about treatment options, and some patients with unilateral lesions chose pharmacotherapy due to a lack of definitive diagnosis, complicating the direct comparison of the two treatments (45).

There are several strengths in this study. First, this study systematically analyzed the differences in efficacy among pharmacotherapy, surgery and minimally invasive treatment for PA, thereby providing a comprehensive basis for clinical decision-making and facilitating physicians’ selection of the most appropriate treatment modality. Second, this NMA integrated data from multiple studies, which further improved statistical power, offered persuasive comparisons regarding efficacy among different treatment options, and provided important indirect evidence, especially in the absence of direct comparison trials. Third, this study explored the advantages of multilevel interventions such as TADR plus RND, revealing that combination therapy may significantly increase antihypertensive efficacy through a dual mechanism of action. This provides more theoretical support and research directions on clinical treatment.

This study has some limitations. First, the distinction between patients with unilateral and bilateral lesions was not always clear, and lack of AVS in some patients may have led to misclassification. Second, most studies provided only short-term follow-up, leaving long-term efficacy and cardiovascular outcomes uncertain. Third, individualized treatment strategies considering comorbidities, age, and gender were not fully explored. Fourth, information on drug dosing was inconsistently reported across studies, which precluded standardized comparisons of pharmacological regimens. In addition, the majority of included studies were conducted in China, raising concerns about regional over-representation and limiting the external validity of our findings. Moreover, as highlighted by the SCOT-PA study (46), considerable heterogeneity exists in diagnostic approaches across centers, contributing to inconsistent diagnosis and heterogeneous populations. Finally, inconsistent subtype diagnosis remains an important limitation. Although the recently proposed Primary Aldosteronism Severity Classification (PASC (47),) could address this issue, its application requires patient-level data, which were not available in this review. Future studies with individual-level reporting are needed to enable formal application of PASC. Similarly, standardized consensus definitions such as the PASO criteria (37) for surgical outcomes and the recently proposed PAMO criteria (7) for medical outcomes could not be applied, as the majority of included studies did not report results accordingly. Future studies with individual-level reporting and standardized outcome definitions are needed to enable formal application of PASC, PASO, and PAMO.

Future studies should further optimize treatment strategies for different subtypes of PA, especially clarify different responses to treatment of unilateral and bilateral lesions, and investigate the impact of combination therapy on long-term prognosis and cardiovascular risk. In addition, given that patients with PA often have concomitant metabolic diseases (e.g., diabetes, obesity), multidisciplinary collaboration is crucial to optimize treatment outcomes and improve quality of life.

## 5 Conclusion

The results of this study showed that for patients with PA, TADR plus RND had the best antihypertensive efficacy in surgical treatment, whereas MRAs plus IRB showed the best efficacy in pharmacological treatment. However, due to the limited number of included studies and the short follow-up time, the results of this study still need to be further validated in future studies.
